# Cytotoxicity of Triterpenoid Alkaloids from *Buxus microphylla* against Human Tumor Cell Lines

**DOI:** 10.3390/molecules21091125

**Published:** 2016-08-26

**Authors:** Shi-Tou Bai, Guo-Lei Zhu, Xing-Rong Peng, Jin-Run Dong, Mu-Yuan Yu, Jian-Chao Chen, Luo-Sheng Wan, Ming-Hua Qiu

**Affiliations:** 1 State Key Laboratory of Phytochemistry and Plant Resources in West China, Kunming Institute of Botany, Chinese Academy of Sciences, Kunming 650201, China; baisitou@126.com (S.-T.B.); zhuguolei@mail.kib.ac.cn (G.-L.Z.); pengxingrong@mail.kib.ac.cn (X.-R.P.); dongjinrun@mail.kib.ac.cn (J.-R.D.); yumuyuan@mail.kib.ac.cn (M.-Y.Y.); jcchen@mail.kib.ac.cn (J.-C.C.); 2 School of Traditional Chinese Medicine, Yunnan University of Traditional Chinese Medicine, Kunming 650500, China; 3 Yunnan Key Laboratory of Natural Medicinal Chemistry, Kunming Institute of Botany, Chinese Academy of Sciences, Kunming 650201, China

**Keywords:** *Buxus microphylla*, triterpenoid alkaloid, cytotoxicity

## Abstract

Three new triterpenoid alkaloids, namely buxmicrophyllines P–R (**1**–**3**), were isolated from the twigs and leaves of *Buxus* *microphylla*. Their structures were elucidated on the basis of NMR and MS spectroscopic analyses. Structurally, compounds **1**–**3** belong to the 9,10-cycloartane type alkaloids. In addition, compound **3** exhibited moderate cytotoxic activities in vitro against HL-60, SMMC-7221, A-549, MCF-7, and SW480 cell lines (with IC_50_ values ranging from 4.51 to 15.58 μM).

## 1. Introduction

**Figure 1:**
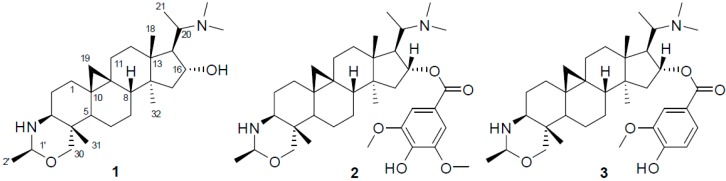
Chemical structures of compounds **1**–**3**.

Plants of the genus *Buxus* are abundant in triterpenoid alkaloids (*Buxus* alkaloids), comprised of more than 140 analogues with a 9,10-cyclopropyl ring system and a degraded C-20 side chain [1]. Some of these alkaloids have been demonstrated to have antimalarial, antituberculosis, anti-HIV, and anticancer activities [2,3,4,5,6,7,8,9]. One of these plants, *B. microphylla* Sieb. et Zucc. (Buxaceae), native to Southern China, is an evergreen shrub and usually planted to beautify the environment [10]. Moreover, twigs and leaves of this plant are used in folkloric medicine for the treatment of tumor, stomachache, hernia, and acute myocardial ischemia [10]. In our continuous search for active alkaloids from this plant [11,12,13], three new triterpenoid alkaloids, namely buxmicrophyllines P–R (**1**–**3**), were isolated from the twigs and leaves of *B. microphylla*. The three compounds (shown in Figure 1) were evaluated for their cytotoxic activities in five human tumor cell lines. Herein, we described the isolation, structure elucidation, and cytotoxicity of these compounds.

## 2. Results and Discussion

Three new triterpenoid alkaloids (**1**–**3**) were obtained by chromatographic separation of the acetone extract of the twigs and leaves of *B. microphylla*.

**Figure 2:**
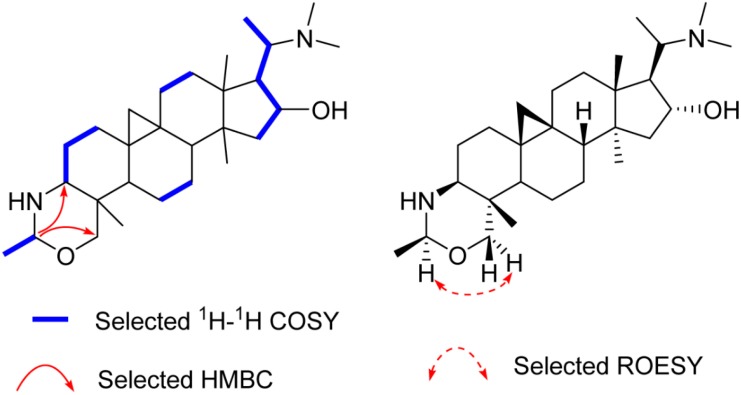
Key 2D correlations of **1**.

Compound **1**, an amorphous powder, gave a molecular formula C_28_H_48_N_2_O on the basis of High Resolution Electrospray Ionization Mass Spectroscopy (HR-ESI-MS) spectrum (*m*/*z* 445.3795 [M + H]^+^, calculated for C_28_H_49_N_2_O, 445.3797). The ^1^H- and ^13^C-DEPT NMR spectra (Table 1 and Supplementary Materials Figures S1–S6) of **1** displayed signals for seven methyls (three tertiary singlets at δ_H_ 0.96, 0.98, and 1.12), nine methylenes (two typical cyclopropyl protons at δ_H_ 0.59 (d, *J* = 4.0 Hz) and 0.33 (d, *J* = 4.0 Hz)), seven methines, and five quaternary carbons. These functionalities suggested that **1** is a typical 9β,10β-cycloartane type triterpenoid alkaloid [12]. Further analysis of the NMR data revealed that the structure of **1** was parallel to that of cyclobuxoxazine [12,14], except for the presence of an additional secondary methyl group (δ_C_ 21.7 and δ_H_ 1.30 (d, *J* = 5.5 Hz)) and a methine group (δ_C_ 85.3 and δ_H_ 4.29) replacing the methylene group (δ_C_ 79.5) at C-1′ in the latter, suggesting that the methyl group should be located at C-1′. This deduction could be further confirmed by the ^1^H-Detected Heteronuclear Multiple Bond Correlation (HMBC) of H-1′ to C-3 and C-30 and the ^1^H-^1^H Correlation Spectroscopy (^1^H-^1^H COSY) of H-2′/H-1′ (Figure 2). The H-1′ proton was assigned as α-oriented by the Rotating-frame Overhauser Enhancement Spectroscopy (ROESY) from H-1′ to Hα-30 (Figure 2). Thereby, the structure of **1** was defined as shown and named buxmicrophylline P.

**Figure 3:**
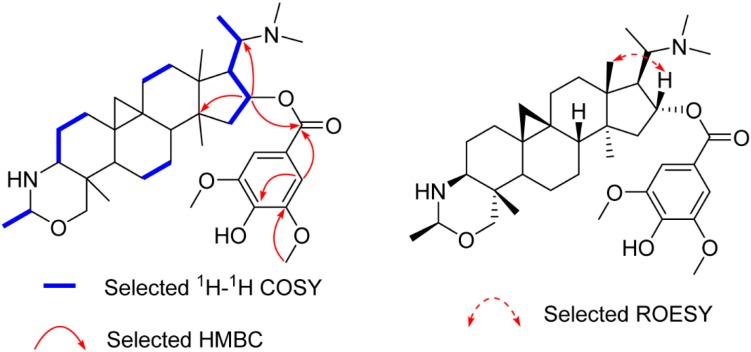
Key 2D correlations of **2**.

Compound **2** yielded the molecular formula C_37_H_56_N_2_O_6_ based on its ^13^C-NMR and the HR-ESI-MS ion peak at *m*/*z* 625.4216 [M + H]^+^ (calculated 625.4212), 180 mass units more than that of **1**, suggesting that it is a syringoylated derivative of **1**. The additional syringoyl group (δ_H_ 7.27 (s, 2H); δ_C_ 165.8, 121.8, 106.5, 146.7, 139.1) and the downfiled shift of C-16 (from δ_C_ 78.9 to δ_C_ 80.4) allowed the location of the syringoyl group at C-16, as confirmed by the HMBC correlations of H-16 (δ_H_ 5.26) to the carbonyl carbon (δ_C_ 165.8) of the syringoyl group, C-20, and C-14 (Figure 3). The β-orientation of H-16 was assigned as that of **1** by ROESY correlations of H-16/H-18 (Figure 3). The structure of **2** (buxmicrophylline Q) was therefore depicted as shown.

**Figure 4:**
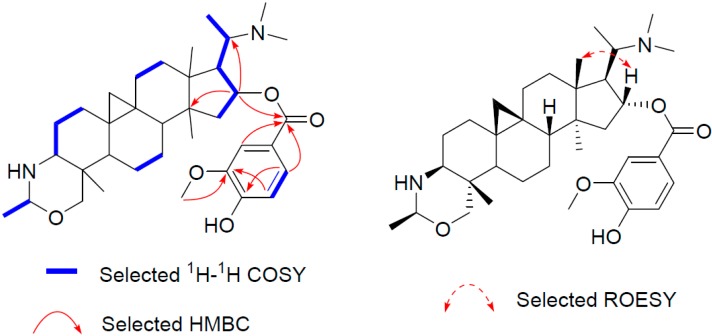
Key 2D correlations of **3**.

Similarly, the structure of compound **3** (buxmicrophylline R), which has the molecular formula C_36_H_54_N_2_O_5_ as determined by the HR-ESI-MS ion peak at 595.4111 [M + H]^+^ (calculated 595.4106), was established by comparing its NMR data with those of **1** and **2**. It turned out that there was a vanilloyl group (δ_H_ 7.60, 7.57, 6.91; δ_C_ 165.8, 123.4, 111.8, 149.5, 146.1, 113.8, 123.7) in **3** rather than a syringoyl group. The location of the vanilloyl group was also at C-16, as confirmed from the HMBC cross peaks of H-16 with the carbonyl carbon (δ_C_ 165.8, Figure 4).

Compounds **1**–**3** were tested for their cytotoxic effects against five human tumor cell lines (Table 2). Compared with the positive control cisplatin, compound **3** displayed the most potent cytotoxicity against MCF-7 cells with IC_50_ values of 4.51 μM. However, the other tested compounds did not exert any cytotoxic effect, even at 40 μM.

## 3. Experimental Section

### 3.1. General Information

Optical rotations were measured with a JASCO P-1020 polarimeter (JASCO Corporation, Tokyo, Japan). UV spectra were obtained using a Shimadzu UV 2401PC instrument (Shimadzu, Tokyo, Japan). Infrared spectra were recorded on a Bruker Tensor-27 instrument (Bruker, Zurich, Switzerland) by using KBr pellets. 1D and 2D-NMR experiments were performed on Bruker AV-400 and DRX-500 instruments (Bruker) with TMS as internal standard. HR-ESI-MS data were acquired on an API QSTAR Pulsar spectrometer (Applied Biosystems, Carlsbad, CA, USA). Column chromatography (CC) was performed on SiO_2_ (200–300 mesh, Qingdao Marine Chemical Group Corporation, Qingdao, China).

### 3.2. Plant Material

The twigs and leaves of *B. microphylla* were collected from Kunming, Yunnan Province, China, in September 2013, and identified by Zong-Yu Wang (Kunming Institute of Botany, Yunnan, China). A voucher specimen (KIB. Bm-20130915) has been deposited at the State Key Laboratory of Phytochemistry and Plant Resources in West China, Kunming Institute of Botany, Chinese Academy of Sciences.

### 3.3. Extraction and Isolation

The chopped, dried plant material of *B. microphylla* (10.0 kg) was extracted three times with acetone (20 L) at room temperature, seven days each time. The filtrate was concentrated under reduced pressure to yield a residue (500 g), which was further suspended in 0.001 N HCl and partitioned with ethyl acetate (EtOAc). The aqueous layer was alkalinized to pH 10.0 with 2 N NaOH followed by exhaustive extraction with CHCl_3_. The CHCl_3_-soluble fraction (120 g) was chromatographed on a silica gel column, eluted with CHCl_3_–MeOH (100:0, 50:1, 20:1, 10:1, 2:1) to give five fractions, A1–A5. Fraction A4 (12 g) was subjected to further silica gel column chromatography using petroleum ether–EtOAc–diethylamine (20:1:1, 10:1:1, 5:1:1, 2:1:1), to yield **1** (20 mg), **2** (4 mg), **3** (5 mg).

*Buxmicrophylline P* (**1**): White amorphous powder; ${\left[\alpha \right]}_{D}^{25}$ +6.8 (*c* = 0.18, MeOH); UV (MeOH) λ_max_ (log ε) 240 (2.13) nm; IR (KBr) ν_max_ 3426, 2938, 2870, 2719, 1634, 1460cm^−1^; ^1^H and ^13^C-NMR data, see Table 1; HRESIMS *m*/*z* 445.3795 (calcd. for C_28_H_49_N_2_O, 445.3797).

*Buxmicrophylline Q* (**2**): White amorphous powder; ${\left[\alpha \right]}_{D}^{25}$ −1.8 (*c* = 0.29, MeOH); UV (MeOH) λ_max_ (log ε) 270 (2.49) nm; IR (KBr) ν_max_ 3429, 2936, 2866, 1708, 1460cm^−1^; ^1^H and ^13^C-NMR data, see Table 1; HRESIMS *m*/*z* 625.4216 (calcd. for C_37_H_57_N_2_O_6_, 625.4212).

*Buxmicrophylline R* (**3**): White amorphous powder; ${\left[\alpha \right]}_{D}^{25}$ −13.7 (*c* = 0.15, MeOH); UV (MeOH) λ_max_ (log ε) 240 (2.56), 265 (2.44) nm; IR (KBr) ν_max_ 3430, 2936, 1710, 1632, 1461, 1292 cm^−1^; ^1^H and ^13^C-NMR data, see Table 1; HRESIMS *m*/*z* 595.4111 (calcd. for C_36_H_5__5_N_2_O_5_, 595.4106).

### 3.4. Cytotoxicity Assay

Compounds **1**–**3** were tested in vitro for their cytotoxicities against five human tumor cell lines (promyelocytic leukemia HL-60, hepatocellular carcinoma SMMC-7721, lung adenocarcinoma A-549, breast cancer MCF-7, and colon adenocarcinoma SW480) by the microculture tetrazolium (MTT) assay. Cytotoxicity evaluations were performed based on the previously described protocol [12], with cisplatin as the positive control. Briefly, after 24 h incubation in 96-well plates, each tumor cell line was exposed to **1**–**3** or positive control at final concentrations of 1, 2, 5, 10, 20, and 40 μM for 72 h. At the end of exposure, 20 μL of 5 mg/mL MTT (Sigma-Aldrich, St. Louis, MO, USA) was added to each well, and the plates were incubated for another 4 h. Then, 100 μL 20% SDS was added, and the plates were further incubated for 12 h. The optical density (OD) was read on a plate reader at 570 nm. IC_50_ values were expressed as concentration of a compound reducing cell growth by 50%. All samples were assayed in triplicate.

## 4. Conclusions 

Phytochemical investigations of *B. microphylla* afforded three new 9,19-cycloartane type alkaloids, buxmicrophyllines P–R (**1**–**3**), together with a known analogue, buxbodine B. In vitro cytotoxicity assay proved that compound **3** exhibited more potent cytotoxic activity against MCF-7 cell line than the positive control, making this compound a potential lead entity for further study. 

## Figures and Tables

**Table 1:** ^1^H-NMR and ^13^C-NMR data of compounds **1**–**3** ^a^.

No.	1		2		3	
	δ_C_, Type	δ_H_	δ_C_, Type	δ_H_	δ_C_, Type	δ_H_
1	33.0, CH_2_	1.26 m; 1.63 m	32.9, CH_2_	1.28 m; 1.61 m	33.0, CH_2_	1.29 m; 1.65 m
2	27.4, CH_2_	1.43 m; 1.53 m	27.1, CH_2_	1.42 m; 1.55 m	27.4, CH_2_	1.46 m; 1.57 m
3	63.3, CH	2.60 m	63.2, CH	2.60 m	63.3, CH	2.60 m
4	37.1, C	-	37.0, C	-	37.1, C	-
5	45.0, CH	1.35 m	44.8, CH	1.35 m	44.8, CH	1.35 m
6	19.9, CH_2_	0.79 m; 1.29 m	19.7, CH_2_	1.84 m; 1.25 m	19.8, CH_2_	0.81 m; 1.26 m
7	25.3, CH_2_	1.11 m; 1.29 m	25.1, CH_2_	1.02 m; 1.25 m	25.2, CH_2_	1.05 m; 1.25 m
8	47.3, CH	1.51 m	46.7, CH	1.60 m	46.8, CH	1.57 m
9	19.1, C	-	18.9, C	-	19.0, C	-
10	25.3, C	-	25.7, C	-	25.8, C	-
11	25.9, CH_2_	1.10 m; 2.00 m	26.0, CH_2_	1.11 m; 1.99 m	26.1, CH_2_	1.10 m; 2.01 m
12	31.5, CH_2_	1.45 m; 1.61 m	32.1, CH_2_	1.58 m; 1.72 m	32.3, CH_2_	1.59 m; 1.74 m
13	44.8, C	-	44.7, C	-	44.9, C	-
14	47.3, C	-	47.7, C	-	47.8, C	-
15	44.4, CH_2_	1.34 m; 1.85 m	44.5, CH_2_	1.41 m; 1.95 m	44.5, CH_2_	1.42 m; 1.98 m
16	78.9, CH	4.05 m	80.4, CH	5.26 m	80.3, CH	5.30 m
17	56.9, CH	1.84 m	56.4, CH	2.27 m	56.6, CH	2.29 m
18	18.7, CH_3_	0.96 s	18.8, CH_3_	1.00 s	18.9, CH_3_	1.03 s
19	30.7, CH_2_	0.59 d (4.0) 0.33 d (4.0)	30.2, CH_2_	0.60 brs 0.33 d brs	30.4, CH_2_	0.63 d (4.0) 0.36 d (4.0)
20	62.4, CH	2.63 m	59.7, CH	2.55 m	59.8, CH	2.57 m
21	9.6, CH_3_	0.87 d (6.4)	9.4, CH_3_	0.84 d (5.8)	9.6, CH_3_	0.86 d (6.4)
30	77.3, CH_2_	3.74 d (10.7) 3.31 d (10.7)	77.2, CH_2_	3.73 d (10.9) 3.28 d (10.9)	77.3, CH_2_	3.75 d (10.7) 3.31 d (10.7)
31	11.4, CH_3_	0.98 s	11.3, CH_3_	0.97 s	11.6, CH_3_	0.99 s
32	20.9, CH_3_	1.12 s	19.5, CH_3_	1.11 s	19.5, CH_3_	1.14 s
1’	85.3, CH	4.29 m	85.1, CH	4.28 m	85.3, CH	4.28 m
2’	21.7, CH_3_	1.30 d (5.5)	21.5, CH_3_	1.28 d (5.4)	21.7, CH_3_	1.31 d (5.4)
N(CH_3_)_2_	40.1, CH_3_	2.09 s	40.4, CH_3_	2.10 s	40.3, CH_3_	2.10 s
OCH_3_	-	-	56.2, CH_3_	3.88 s	56.0, CH_3_	3.93 s
OSyr ^b^ (OVan) ^b^	-	-	165.8, C 121. 8, C 106.5, CH 146.7, C 139.1, C	7.27 s	165.8, C 123.4, C 111.8, CH 149.5, C 146.1, C 113.8, CH 123.7, CH	7.60 brdd 7.57 brd 6.91 d (8.3)

^a^ δ in ppm, *J* in Hz, 600 MHz for ^1^H and 150 MHz for ^13^C, in CDCl_3_. ^b^ OSyr stands for syringoyl group; OVan stands for vanilloyl group.

**Table 2:** Cytotoxic activities of compounds **1**–**3** with IC_50_ values (μM).

Compounds	Cell Lines				
	HL-60	SMMC-7721	A-549	MCF-7	SW480
**1**	>40	>40	>40	>40	>40
**2**	>40	>40	>40	>40	>40
**3**	13.88	15.58	11.76	4.51	13.71
Cisplatin	1.22	4.48	6.18	15.23	11.99

## Supplementary Materials

Supplementary materials can be accessed at: http://www.mdpi.com/1420-3049/21/9/1125/s1.
