# Supplementary material for: Cytotoxicity of Triterpenoid Alkaloids from Buxus microphylla against Human Tumor Cell Lines
Source: Molecules. 2016 Aug 26;21(9):1125. doi: 10.3390/molecules21091125 (PMC6273435; doi:10.3390/molecules21091125)
Supplement: Supplementary file 1 [file molecules-21-01125-s001.pdf]

Supplementary Materials: Cytotoxicity of Triterpenoid Alkaloids from *Buxus microphylla* against Human Tumor Cell Lines

Shi-Tou Bai, Guo-Lei Zhu, Xing-Rong Peng, Jin-Run Dong, Mu-Yuan Yu, Jian-Chao Chen, Luo-Sheng Wan, and Ming-Hua Qiu

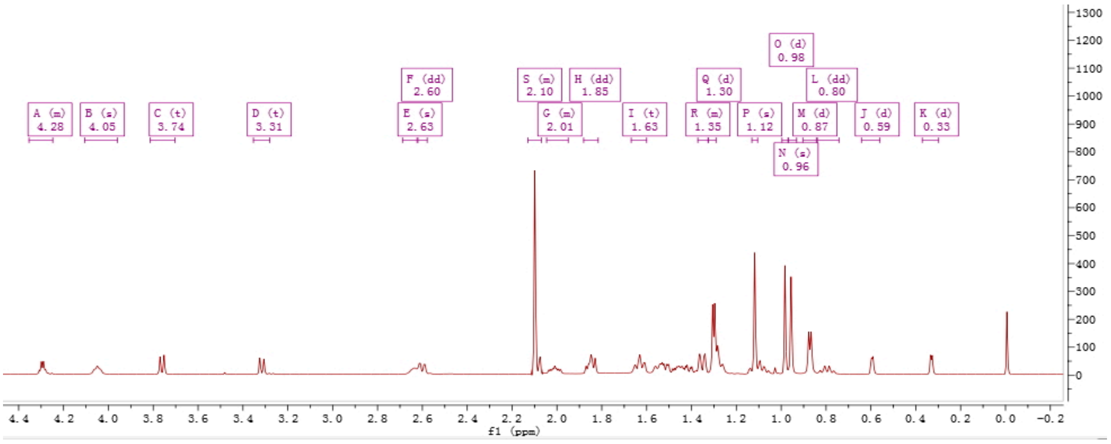

Figure S1. ^{1}H-NMR spectrum of compound **1** (600 MHz, in $CDCl_3$).

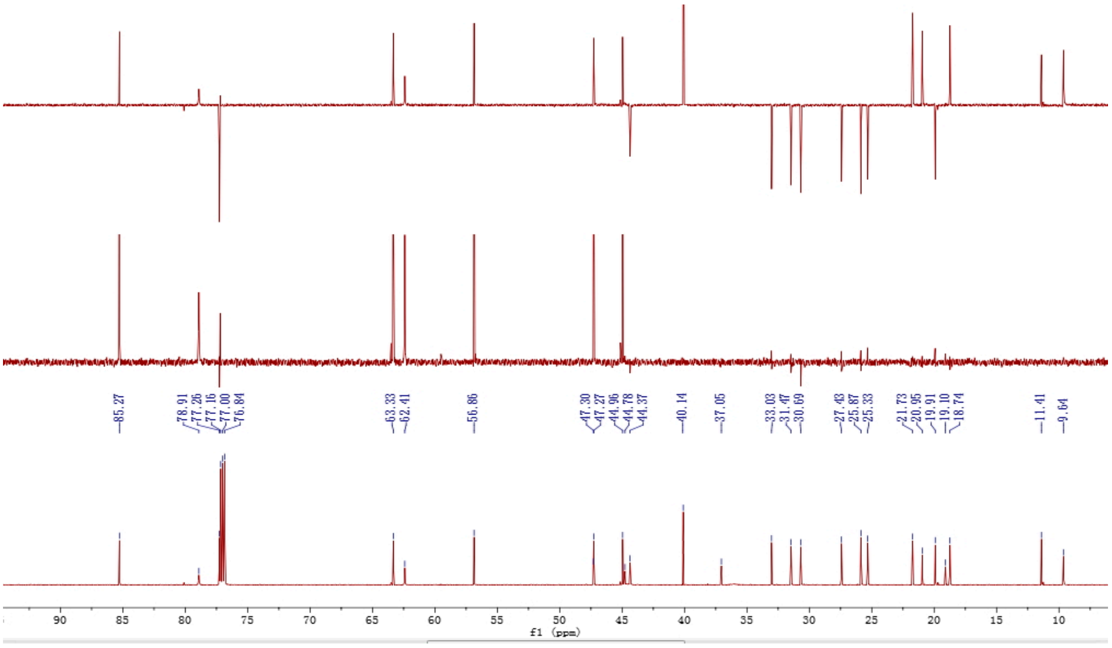

Figure S2. ^{13}C-DEPT NMR spectrum of compound **1** (150 MHz, in $CDCl_3$).

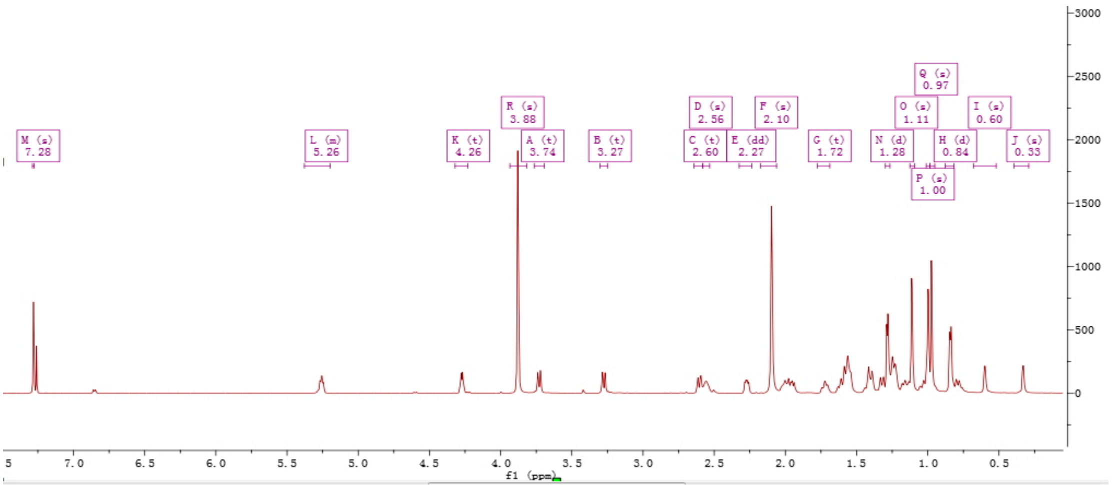

Figure S3. ^{1}H-NMR spectrum of compound **2** (600 MHz, in $CDCl_3$).

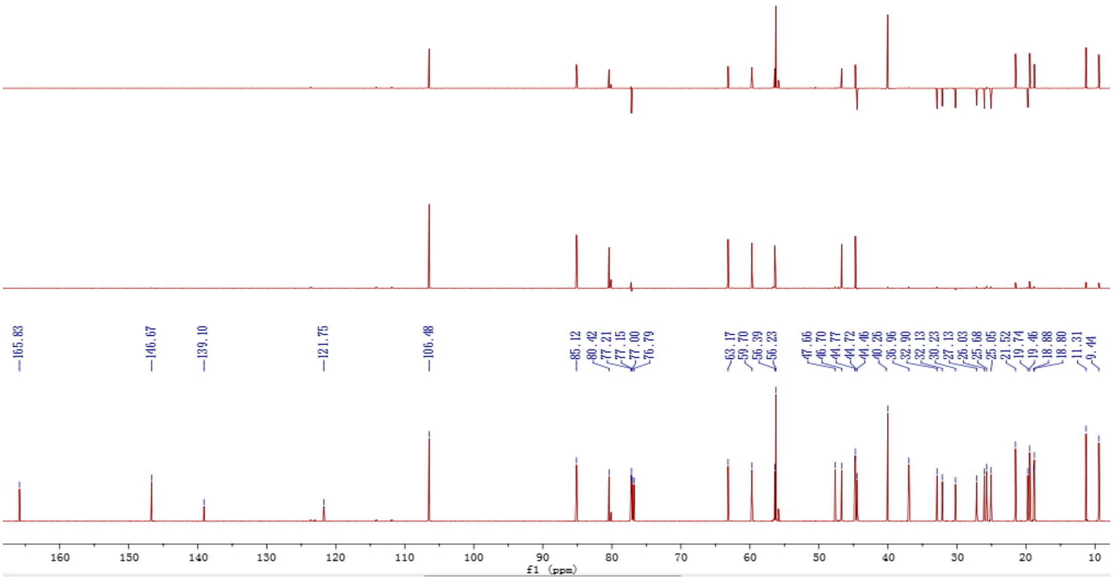

Figure S4. ^{13}C-DEPT NMR spectrum of compound **2** (150 MHz, in $CDCl_3$).

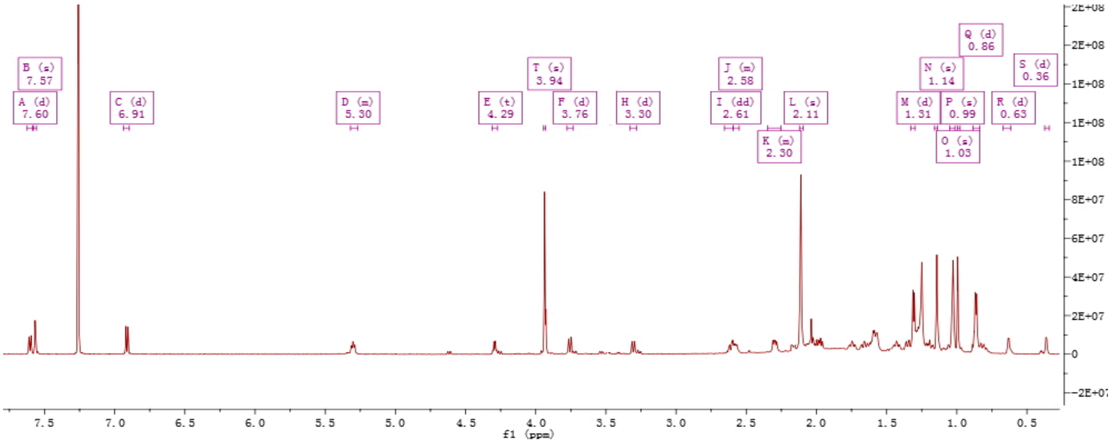

Figure S5. ^{1}H-NMR spectrum of compound **3** (600 MHz, in $CDCl_3$).

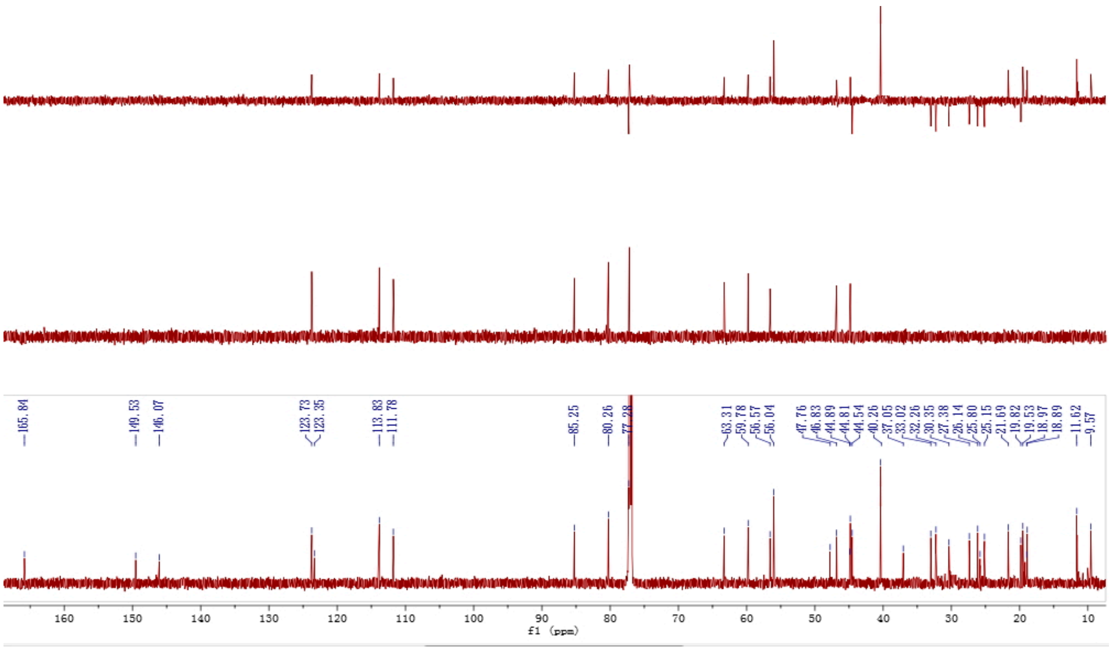

Figure S6. ^{13}C-DEPT NMR spectrum of compound **3** (150 MHz, in $CDCl_3$).
